# Development of an information system and mobile application for the care of type 2 diabetes patients at the primary care level for the health sector in Mexico: study protocol for a randomized controlled, open-label trial

**DOI:** 10.1186/s13063-022-06177-0

**Published:** 2022-04-04

**Authors:** Noël C. Barengo, Leticia Manuel Apolinar, Norma A. Estrada Cruz, José E. Fernández Garate, Roberto A. Correa González, Paula A. Diaz Valencia, Cecilia Alicia Cinco Gonzalez, José Alberto Gómez Rodriguez, Nelly Cisneros González, On behalf of the SANENT Study Group, Maria L. Arellano Flores, Mercedes E. Ledesma Muñoz, Diana A. Gonzalez Sotelo, Oscar M. Davila Maldonado, Jhoana G. Gomez Garcia, Francisco J. Laureano Hernandez, Julio Eduardo Zarazua Jimenez, Brenda A. Pulido Garcia, Hector Rodriguez Vazquez, Alexis A. Ramirez Dorantes, Liliana A. Gonzalez Fierro, Juan C. Hernandez Hernandez, Jorge Zenil Perez

**Affiliations:** 1 Herbert Wertheim College of Medicine & Robert Stempel College of Public Health, Florida International University, Miami, FL USA; 2 Unidad de Investigación Médica en Enfermedades Endocrinas, Instituto Mexicano del Seguro Social, Mexico City, Mexico; 3 Consejo de Salubridad General, Mexico City, Mexico; 4 Facultad de Salud Pública, Universidad de Antioquia, Medellín, Colombia

**Keywords:** Primary care, mHealth, Type 2 diabetes, Control, Treatment compliance, Telemedicine

## Abstract

**Background:**

Providing optimal care for type 2 diabetes (DM2) patients remains a challenge for all healthcare systems. Patients often encounter various barriers in adhering to self-management programs due to lack of knowledge and understanding of self-care activities, lack of individualized and coordinated care, inconvenient and costly education sessions, and poor patient-provider communication. Mobile technologies such as cell phones/smartphones, handheld tablets, and other wireless devices offer new and exciting opportunities for addressing some of these challenges. The purpose of this study is to compare a diabetes management strategy using an information board and a mobile application versus standard care in patients with uncontrolled DM2.

**Method:**

The SANENT (Sistema de Análisis de Enfermedades No Transmisibles) trial is a primary care-based, prospective, two-arm, randomized controlled, open-label, blinded-endpoint study. We aim to recruit 1440 DM2 patients during a period of 6 months until the requested number of participants has been achieved. The total length of the intervention will be 1 year. Both men and women treated for DM2 with an HbA1c > 8.5% and ≥ 20 years of age are eligible to participate in the study. The primary outcome of the study is improved diabetes control measured by changes in HbA1c in the study participants. HbA1c will be measured at baseline, 3-month, 6-month, 9-month, and 12-month follow-up visits in all participants. The main analysis will be based on the intention-to-treat principle. The primary endpoint of the study will be the change in HbA1C within the groups and the differences between the groups. This will be assessed by a repeated measurement approach based on mixed models which contain both fixed effects and random effects.

**Discussion:**

The overall goal of this project is to contribute to the evidence for the use of mobile technology to improve the treatment and regulation of poorly controlled DM2 patients living in Mexico. Our proposed project will show how mobile health technology tools can be used in the treatment of patients with uncontrolled DM2 in primary health care in a Latin American population, and particularly how they could help diabetes patients take better care of themselves.

**Trial registration:**

ClinicalTrials.gov, US National Institutes of Health NCT04974333. Prospectively registered on July 13, 2021. Protocol version number 1, dated August 15th, 2021.

## Background

Type 2 diabetes mellitus (DM2) is one of the fastest-growing public health problems in developed and developing countries and imposes a large financial burden on healthcare systems [1]. The International Diabetes Federation has estimated that the number of adults with diabetes mellitus in Mexico is expected to rise from 12.8 million in 2019 to over 22 million by 2045 of whom more than 95% would have had DM2 [2]. DM2 is a condition that is difficult to treat. Cardiovascular, cerebrovascular, and peripheral vascular diseases are the major complications in patients with DM2 [3]. Moreover, research has shown that patients with DM2 are likely to have a 12–14 years shorter lifespan compared with people without DM2 [4, 5]. Annual health care costs of a patient with DM2 are approximately two to three times higher compared with a person of similar age and sex without diabetes. It has been estimated that approximately 80% of the cost of diabetes to health services in developed countries is spent on complications [6, 7]. With projected increases in the prevalence of diabetes in Mexico and costs arising from the long-term complications incurred by this condition, primary healthcare providers have started to identify self-management interventions that improve diabetes care and patient-related outcomes [8].

Current research has shown that mobile health interventions in patients with DM2 may reduce HbA1c by 0.8% compared with standard care [8–10]. In addition, scientific evidence has revealed a 37% reduction in microvascular complications for each 1% reduction in HbA1c level and an increase in quality-adjusted life years [11–14]. A British study concluded that around 789,000 microvascular and 81,000 macrovascular events could be avoided in the UK among DM2 patients with a 0.8% HbA1c reduction [15].

A recent review showed that the use of mHealth technology in the clinical care of patients with DM2 can result in improved treatment outcomes [16]. They also highlighted that a mHealth intervention need not be restricted to shot messages (SMS) alone. Mobile health technologies have the capacity to grant fast access to information and aid in instant communication for patients with DM2 and healthcare professionals [17]. One third of the current studies were in North America and one third in Asia [18]. Only few studies included DM2 patients in South- America (5%). Moreover, the study setting varied a lot in previous studies with only a 24% of the scientific studies carried out in the primary care sector or specialized outpatient clinics (14%). The mobile health interventions used in previous international studies were also diverse in nature and utilized a wide range of technological innovations such as text messaging (SMS), mobile applications as well as secure websites/web-portals that could be accessed through the diabetes patients' mobile device [8]. Thus, more information is needed on the benefit of mobile health technologies in clinical care settings and whether combining different technological innovations may provide additional benefits.

Therefore, this trial will compare a diabetes management strategy using an information board and a mobile application versus standard care in patients with uncontrolled DM2 within the primary health care system in Mexico.

## Methods

### Trial design

The SANENT (Sistema de Análisis de Enfermedades No Transmisibles [Analysis System for Non -Communicable Diseases]) trial is a primary care-based, prospective, two-arm, randomized controlled, open-label, blinded-endpoint study. This trial aims to recruit 1440 DM2 patients during a period of 6 months until the requested number of participants has been achieved. The total length of the intervention will be one year. The SANENT trial protocol is presented according to Standard Protocol Items: Recommendations for Interventional Trials (SPIRIT) recommendations [19].

### Trial setting

This trial will be conducted within primary health care practices in three states (Colima, Tlaxcala, and Guanajuato) of Mexico. These states were selected because they are some states with a high prevalence of diabetes in México [20–22] and have implemented an Electronic Health Record System.

### Patient and public involvement

During the planning phase of the study, a pilot project was carried out evaluating the design and applicability of the information board and mobile application in 31 DM2 patients. The final intervention was then modified according to the qualitative data received from the patients. We also involved primary healthcare clinicians in the development and evaluation of the information. The results of the study will be made available to all trial participants and participating general practices.

### Sample size calculations

Considering a difference in HbA1c of 1.0% between the intervention and the control group, a standard deviation of 1.5%, a power of 90%, and an alpha of 0.05 (two-sided tests) with a drop-out rate of 25% during the study, a minimum of 126 will be needed in total in each of the three states. As each state where this study will be carried out has a different healthcare provider, we decided to stratify the analysis according to participating state to see whether the intervention works regardless of the healthcare provider. Thus, 126 participants will be recruited in each state. Thus, the total number of patients will be 378. The reason for testing the intervention in each state differently is as the patients belong to different healthcare providers. There may be differences between these systems. We would like to see whether the intervention works within each of the healthcare provider’s systems.

### Screening and recruitment

The participating healthcare centers were selected because they have the most recent version of the Electronic Health Record System that allows linking clinical information to the mobile app and the information board used in this study. These centers count with the necessary health information system infrastructure. Each of these centers takes care of the clinical control of diabetes patients. The primary healthcare workers of the participating centers will screen their DM2 patient lists and will invite eligible patients. Patients will receive an invitation letter and a leaflet with general information about the study. Eligible patients may also be contacted by phone, emails, or text by the healthcare professionals. Patients will be enrolled for screening over a 9-month period (t −2). During the first visit of the screening phase (time point t −1; Table 1), the eligibility criteria and medical record will be revised by the healthcare professionals. In case the patient is potentially eligible, they will be invited to a HbA1c measurement to complete the second eligibility assessment (t −1). Once the diagnosis of uncontrolled DM2 has been made, the patient will be asked to sign a written consent. In the next phase (t 0) the participants will be randomly allocated to either the intervention or control group (t 0). At the same time, all other baseline measurements and laboratory tests will be conducted (t 0).

**Table 1:**
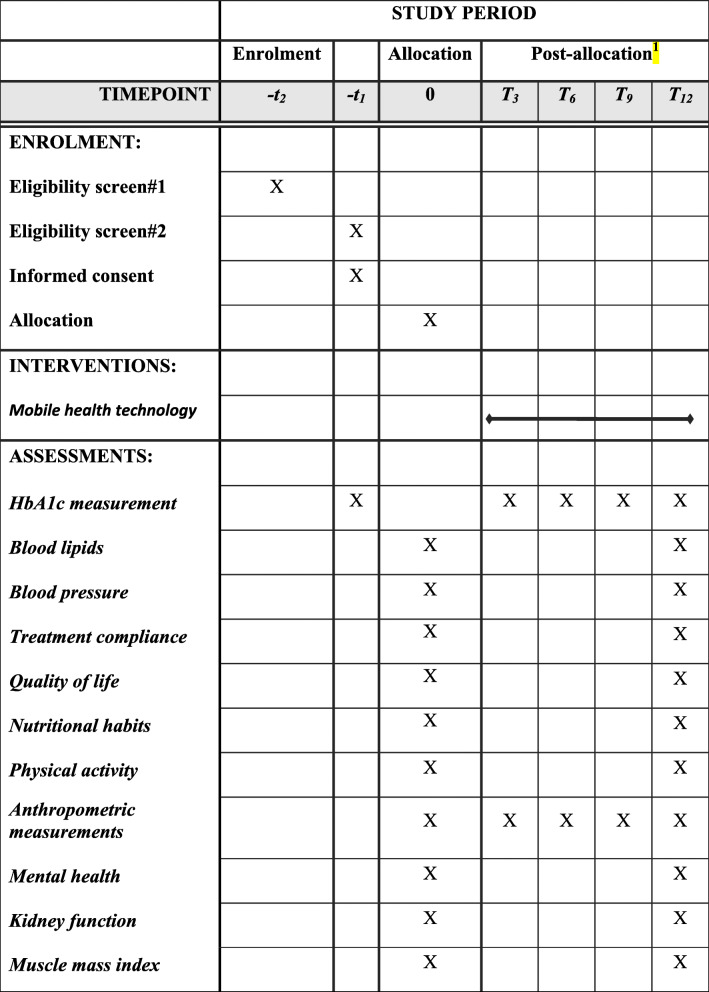
The schedule of enrolment, interventions, and assessments in the SANENT trial

^1^time-points after randomization

### Inclusion and exclusion criteria

The inclusion and exclusion criteria for patient enrolment are presented in Table 2. Both men and women treated for DM2 with an HbA1c> 8.5% and ≥20 years of age are eligible to participate in the study. Eligible patients need to be signed up for diabetes treatment and control in one of the participating primary healthcare practices. Participants need to have access to a mobile phone or, alternatively, have a family member who will help them in sending, understanding, and retrieving messages and information provided in Spanish language through the mobile application.

**Table 2:** Inclusion and exclusion criteria for patient enrolment

Inclusion criteria	
- Men and women with DM2 - Age at least 20 years old - HbA1c > 8.5% - Have access to a smartphone or have a family member over 18 years of age with a smartphone and will help in sending, understanding, and retrieving messages and information - Assigned for diabetes treatment and control to one of the participating primary healthcare centers - Sign informed consent to participate in the study	
**Exclusion criteria**	
- Pregnancy, within 3 months post-partum or planning pregnancy during the trial - Breastfeeding - Serious medical condition (i.e., dialysis) - Not be a permanent resident of the participating states	

Participants who are pregnant, within 3 months postpartum or planning pregnancy during the trial; are breastfeeding; have a serious medical condition that, in the opinion of the investigator, makes them ineligible (i.e., dialysis treatment); have been admitted to hospital within the last 3 months for hyperglycemia or hypoglycemia; or are not permanent residents of the states the study is conducted are not eligible to participate in the study.

### Random allocation

This trial will use a parallel-group design, randomizing patients to either the intervention or the control arm by a computer-generated sequence with an allocation ratio of 1:1. The randomization of the study participants will be done after having provided consent and when all baseline assessments have been completed to minimize reporting and selection bias.

Random allocation will be done using a validated secure web-based randomization operated by a data manager, not involved in the patient recruitment, located at the Universidad Nacional Autonoma de Mexico. This will ensure concealment of the treatment sequence up to the allocation. The treatment sequence will be generated by a computer-generated sequence of random numbers. The block randomization method will be used. Allocation will be carried out with an algorithm to ensure groups are balanced for important baseline prognostic and other factors: study site, age (< 65/≥65 years), sex (male/female), duration of diabetes (< 5 years/≥5 years) and number of medications (< 5/≥5) which are considered as a key prognostic variable for the primary outcome of this trial. The treatment allocation codes will be concealed in sequentially numbered envelopes that will be opened each time a patient will be enrolled.

### Blinding

Because the SANENT trial is an open-label study, neither the primary healthcare physicians nor patients can be blinded to the patients’ allocation to either intervention or control arm. Thus, blinding will apply only for the study endpoints. To guarantee the blind assessment of the primary endpoint of the study (HbA1c reduction), the laboratory personnel involved in its measurement will be blinded to the patient’s allocated treatment strategy. Also, the statistician analyzing the data will be blinded in regard to the patients’ allocated groups.

### Interventions

Participants assigned to the intervention group will use a mobile application on their smart phones that will assist them with their diabetes control, provide and update information on their clinical history, offer a monitoring of their nutritional and physical activity habits as well as anthropometric measurements, provide recommendations how to improve on nutritional habits and physical activity behavior, and assist in planning and reminding on clinical appointments. The mobile health application was tested and evaluated in a pilot project in 31 DM2 patients as part of this project. Based on the feedback received, the mobile application was revised and finalized to be sued in this project.

The content and description of the patient mobile health application are presented in Table 3.

**Table 3:** Description of the patients’ mobile health application.

Subject	Description of content
Patient profile	Patient’s personal information, as well as information regarding clinical history, laboratory measurements, and current pharmaceutical treatment.
My state of health	Information about age, body weight, height, body mass index, glucose, total cholesterol, triglycerides, blood pressure, and glycated hemoglobin (HbA1c).
My measurements	Information about serum glucose, blood pressure, heart rate, respiration frequency, and oxygen saturation.
My studies	Information is classified into three analysis sections. A) Health center laboratory; Serum glucose, total cholesterol, triglycerides, glycated hemoglobin (HbA1c), creatinine and urea. B) Alternate laboratory (Lab Salud Digna); Serum glucose, total cholesterol, HDL cholesterol, LDL cholesterol, triglycerides, HbA1c, serum creatinine, serum albumin and urea. C) Complementary studies: information about of ECG, check-ups to assess diabetes complications (retinopathy, nephropathy, neuropathy) study visits.
My medications	Record of pharmacological treatment with its doses.
My body	interactive module monitoring nutritional habits, physical activity, patients’ self-perceived mood, and taking care of my feet. This module also contains a section that allows the patient to view changes and trends in their anthropometric measurements.
My recommendations	Provides recommendations on healthy eating habits, physical activity, mental health, and taking care of my feet.
Medical appointment	Information on upcoming clinical appointments (clinical control, laboratory tests).
Contact	Interactive module allowing patients to provide questions and comments about the mobile application.

The participating physicians will have access to the patient’s information through a mobile information tablet. That specific tool will allow the physician to view and consult all patient information of the patient’s mobile application. It will use a Java programming language, having an exchange of information through the Apache Web Server and from the Tomcat Application Server in a Java environment inbuilt on the SQL Server manager. Thus, the participating physician will have direct access to all clinical information, visits, lifestyle changes, and cardio-metabolic control of the DM2 patient. The main idea is that the mobile information board will help the physician to monitor better the treatment and control of his patients.

Participants assigned to the control arm will continue their diabetes care without mobile health technology assistance. This comparer was chosen as it is the current standard of care in the Mexican primary healthcare system.

The duration of the intervention will be 12 months. Each patient will be invited to follow-up visits and measurements every 3 months (month 3, month 6, month 9, and month 12).

### Strategies to improve compliance to the intervention

The research team has developed several strategies to improve compliance during the intervention:

1. All study participants will be informed and reminded about all testing arrangements, intervention routines, benefits, and potential harms to make them fully understand the importance of their participation in the study.
2. All study participants will perform the intervention routine under the supervision of qualified medical personnel.
3. The study coordinator will maintain contact with all study participants throughout the study and remember them about all scheduled appointments. If an appointment will be missed, the trial coordination team will try to contact the study participant and assist with complying with the scheduled visits.

### Relevant concomitant care permitted or prohibited during the trial

The study participants will be asked not to take part in any other programs related with clinical control of their diabetes during the proposed trial. In addition, participants are prohibited from taking any other treatments as part of their routine medical care.

### Outcomes

The primary endpoint of the study will be the change in HbA1C within the groups and the differences between the groups. HbA1c will be measured at baseline, 3-month, 6-month, 9-month, and 12-month follow-up visits in all participants. The primary time-points for HbA1C are the 6- and 12-month measurements (Table 1).

The secondary outcomes of the study include differences in the mean cholesterol, LDL cholesterol, HDL cholesterol, systolic and diastolic blood pressure, triglycerides, microalbuminuria, waist circumference, body mass index, and muscle mass index. The laboratory measurements will be performed at the beginning of the intervention and at month 12 (Table 1). The anthropometric measurements will be done every 3 months. In addition, secondary outcomes will be treatment compliance measured by the Morisky score [23], quality of life using the Short Form 36 Health Survey Questionnaire (SF-36) [24], nutritional habits assessed by the ENSANUT questionnaire [25], and physical activity by the international physical activity questionnaire [26]. In addition, anxiety and mental health will be evaluated using the Beck questionnaires [27, 28]. The questionnaires will be applied at baseline and at 12 months of the intervention (Table 1).

Finally, process indicators related to the use of mobile application and the information board (usage time and frequency, user satisfaction among others) will also be assessed.

### Data analysis plan

All data collected locally at each enrolling unit will be recorded into an electronic database. Clinical data to be input into the database include demographic information, results on blood and urine laboratory tests performed in each center, ongoing treatment (dose and medication), as well as information on adverse events during follow-up of the trial. The quality of data obtained will be validated by expert personnel at each study site.

#### Data management

All data will be entered twice (double data entry) and checked by two independent administrators to improve the quality and accuracy of data. The data obtained in this study will be stored at the Consejo de Salubridad General of Mexico in an electronic database. Only the data administrators and statistician will have access to the stored data. They will also be responsible for data backup. All data will be archived at the Consejo de Salubridad General of Mexico for a minimum of 5 years from the study end.

#### Confidentiality

The information collected will be used for research purposes and be analyzed without the personal identification of the participants. None of the personal information of enrolled participants will be shared or released.

#### Descriptive analysis

The first step in the data analysis will be a descriptive analysis that will summarize continuous variables using mean value, median, standard deviation, and interquartile range. Categorical data will be described using absolute and relative percentages. The incidence of adverse events will be tabulated by the treatment group.

#### Primary and secondary endpoint analysis

The primary endpoint of the study will be the change in HbA1c between the intervention and the control group. This will be assessed by a repeated measurement approach based on mixed models which contain both fixed effects and random effects. These models are likelihood-based approaches in the presence of non-ignorable missing data (e.g., missing at random). the main purpose will be to determine whether the within-person changes over time vary across levels of one or more between-person factors (e.g., blood pressure measurement technique and for the same covariates included in the model for coprimary endpoints). Exploratory analysis will consist of profile graphs and Pearson’s correlation matrix. The Mauchly test will be applied to verify the fulfillment of the Huynh-Feldt condition. A mixed linear model will be used. To select the most appropriate covariance matrix, the Alkaike AIC or the Swartz BIC criteria will be used. The models will be controlled for age, sex, and time with the diagnosis of diabetes. No subgroup analysis will be conducted. Also, interim analyses will not be performed in this study.

#### Process evaluation

Qualitative-quantitative analysis will be used to summarize the experience of the study participants and health professionals in their use of and implementation of the mobile application and the information board. Interviews with up to 30 participants in each state will take before and after the 6-month follow-up. Additionally, up to 10 healthcare professionals will be invited to share their feedback and experiences of the intervention and their participation in the study.

Participants who consent to taking part in the qualitative study will be purposefully selected by characteristics including age, gender, and length of time of diabetes and invited to share their views on mobile application and to provide insight into how it was implemented in daily life and identify issues around potential compliance. Healthcare staff from participating healthcare will be invited to participate in focus groups or qualitative interviews to share their experience on how the intervention was implemented in routine clinical care and their experience in using the information board.

#### Methods in analysis to handle protocol non-adherence and any statistical methods to handle missing data

The data will be analyzed according to the original group allocation using intention-to-treat analysis. If any participant withdraws from the trial, multiple imputation method will be used to adjust for the missing data.

### Monitoring

To monitor our study, a trial Steering Committee and a Data Monitoring Committee will be appointed. The Trial Steering Committee is taken on by the General Health Council of Mexico which consists of seven members led by Secretary Dr Jose Ignacio Santos. The Steering Committee will monitor the study procedures and make sure that the trial is being carried out according to the approved study protocol. The Data Monitoring Committee will comprise four members including one researcher from the Universidad Nacional Autonoma de Mexico. All members of the Data Monitoring Committee will have clinical and research expertise as well as expertise in statistical analysis and data management. They will regularly perform quality checks of the data received by the electronic data collection system. The Data Monitoring Committee will also review compiled adverse event data at periodic intervals and report to the ethics committee any safety concerns and recommendations for suspension or early termination of the trial. Interim analysis will not be conducted in this study.

Finally, the trial Steering Committee will meet on a weekly basis to report on the progress of the trial. In addition, the trial Steering Committee and the Data Monitoring Committee will meet monthly to review the advances of the study.

This study does not plan to conduct an auditing of trial conduct.

### Criteria for discontinuing or modifying allocated intervention

Study participants can withdraw from the study if they make such a request, if they develop a serious disease (i.e., stroke and acute ischemic heart attack), if the principal investigator considers the participant as unsuitable to continue the study, or if the study participant reports serious adverse events during the study.

### Ethical considerations

The study protocol was approved by the Ethical Committee in Research, Secretaria de Salud, State of Colima-Mexico approved this study: 2021/1/CR/BS/EPI/162. Each study participant will sign informed consent. Study participants can withdraw from the trial at any time. Participants can also choose to pause or stop receipt of text messages or e-mails by written notice or contacting the trial office by phone. Information on adverse events will be registered on a regular basis. The occurrence of serious unexpected adverse events related to the intervention will be determined by the principal investigator and reported according to local procedures. The trial will be conducted according to the principles of the Declaration of Helsinki and in accordance with other relevant national guidelines, regulations, and acts and using Good Clinical Practice guidelines. Lilly Global herald partnership program is sponsoring the trial. Finally, this is an investigator-generated study and will be performed in full independence of the study sponsor from any other funding body.

#### Plans for communicating important protocol amendments to relevant parties

Any important modifications of the study protocol, informed consent form, will be reported to all investigators, trial participants, trial registry, and ethics committee that approved the study.

### Dissemination plan

The results will be reported in conferences or peer-reviewed journals. The results will also be shared with participants, healthcare professionals, and healthcare providers. The results will also be disseminated to the public through social and news channels.

## Discussion

Mobile health technologies such as cell phones/smartphones, tablets, and other wireless devices provide new and innovative opportunities for remote monitoring of DM2 patients and delivery of clinical care through for instance text messaging, internet browsing, emails, and educational videos. Thus, Mobile technologies may be used to support blood glucose monitoring, measurement of daily physical activity and nutritional habits, and other activities that can facilitate diabetes self-management and enhance patient-provider communication [29, 30]. Face-to-face consultations for managing diabetes are costly and seldomly support self-management by patients. Therefore, the mobile health technologies developed in this project will address DM2 treatment and clinical control reducing the costs for the primary healthcare system. Moreover, scientific evidence has revealed that interventions using mobile health technologies targeting health behavior changes aligned with self-determined goals lead to improved physical and mental health outcomes [31].

Typical use of mobile health technologies for the treatment of diabetes has been focused on mobile and web-based applications [32–46]. A recent review of 15 systematic reviews summarized that mobile health technologies interventions improve glycemic control (HbA1c) compared with standard care or other approaches by as much as 0.8% for patients with DM2 and 0.3% for patients with DM1, at least in the short-term (< 12 months) [8]. One of the very few studies using mobile health technologies conducted among the Hispanic population revealed that the use of a simple, low-cost text-messaging (up to three motivational, educational, and/or call-to-action text messages per day over 6 months) program was highly acceptable in a sample of high-risk, Hispanic DM2 patients in Texas and led to greater improvement in glycemic control compared with usual care [47]. None of the previous work has combined educational strategies with mobile technologies to improve treatment compliance and self-management. Latin American DM2 patients have specific needs, and this should be reflected in diabetes mobile health applications designed for this population. Existing research suggests that many Latin American DM2 patients do not possess sufficient diabetes knowledge or self-awareness to fully benefit from the most prevalent functionalities offered by the most popular diabetes mobile applications [47]. Therefore, it has been recommended that developers incorporate more basic features such as diabetes education, reminders to check blood glucose levels or to take medications, Spanish language interfaces, and glucometer connectivity, which are relatively underrepresented in the most popular diabetes applications currently available in Spanish. The mobile tools that have been developed for this project incorporate all these components to achieve the objectives. The main innovation of this prosed project is that we are interested in showing how mobile health technologies can improve motivation in a primary health care setting in a Latin American population of a middle-income country and particularly how these strategies could help patients take better care of themselves and help patients to take better decisions about the management of their health condition.

The software system will also estimate the best set of recommendations and guidelines to better achieve the objectives established with their healthcare specialists. To our knowledge, this is one of the first attempts to develop and test a mobile health technology for improving the treatment and control of DM2 patients in a large group of DM2 patients within the primary care setting in Mexico. If successful, this will result in a decrease in long-term micro- and macrovascular complications, health-related and economic burden for the health care system, and improving the quality of life of DM2 patients. Furthermore, the intervention provided by mobile health technology may revolutionize the treatment and control of DM2 within Mexico providing scientific evidence of the benefits of using mobile health technology in primary healthcare. As the usage of smart phones is on the constant rise even among lower socio-economic groups, a mobile tool with personalized strategies to educate patients and increase treatment adherence may offer a cost-effective response to the increased healthcare costs due to face-to-face meetings with health professionals.

## Trial status

The start date is September 20th, 2020, and the entire study is likely to be completed by 31 October 2023. Recruitment will start from 20 August 2021 and will be completed by April 12, 2022. The trial is likely to be completed by September 2022.

## Data Availability

After the study completion, the final dataset will be available for investigators. It will also be available for the public from the corresponding author on reasonable request.

## Abbreviations

DM1: Type 1 diabetes
DM2: Type 2 diabetes
ECG: Electrocardiogram
ENSANUT: National Survey on Health and Nutrition
HbA1c: Hemoglobin A1c
HDL: High-density lipoprotein
LDL: Low-density lipoprotein
SANENT: Sistema de Análisis de Enfermedades No Transmisibles [Analysis System for Non-Communicable Diseases]
SF-36: Short Form 36 Health Survey Questionnaire
SQL: Structured Query Language
SPIRIT: Standard Protocol Items: Recommendations for Interventional Trials
